# The Stringent Response and Cell Cycle Arrest in *Escherichia coli*

**DOI:** 10.1371/journal.pgen.1000300

**Published:** 2008-12-12

**Authors:** Daniel J. Ferullo, Susan T. Lovett

**Affiliations:** Department of Biology and Rosenstiel Basic Medical Sciences Research Center, Brandeis University, Waltham, Massachusetts, United States of America; Stanford University, United States of America

## Abstract

The bacterial stringent response, triggered by nutritional deprivation, causes an accumulation of the signaling nucleotides pppGpp and ppGpp. We characterize the replication arrest that occurs during the stringent response in *Escherichia coli*. Wild type cells undergo a RelA-dependent arrest after treatment with serine hydroxamate to contain an integer number of chromosomes and a replication origin-to-terminus ratio of 1. The growth rate prior to starvation determines the number of chromosomes upon arrest. Nucleoids of these cells are decondensed; in the absence of the ability to synthesize ppGpp, nucleoids become highly condensed, similar to that seen after treatment with the translational inhibitor chloramphenicol. After induction of the stringent response, while regions corresponding to the origins of replication segregate, the termini remain colocalized in wild-type cells. In contrast, cells arrested by rifampicin and cephalexin do not show colocalized termini, suggesting that the stringent response arrests chromosome segregation at a specific point. Release from starvation causes rapid nucleoid reorganization, chromosome segregation, and resumption of replication. Arrest of replication and inhibition of colony formation by ppGpp accumulation is relieved in *seqA* and *dam* mutants, although other aspects of the stringent response appear to be intact. We propose that DNA methylation and SeqA binding to non-origin loci is necessary to enforce a full stringent arrest, affecting both initiation of replication and chromosome segregation. This is the first indication that bacterial chromosome segregation, whose mechanism is not understood, is a step that may be regulated in response to environmental conditions.

## Author Summary

Management of cell growth and division in response to environmental conditions is important for all cells. In bacteria, nutritional downturns are signaled by accumulation of the nucleotide ppGpp. Amino acid starvation causes a programmed change in transcription, known as the “stringent response”; ppGpp also causes an arrest of cell cycle in bacteria, whose mechanism has not been thoroughly investigated. Here, we show that *E. coli* cells, when the stringent response is in effect, complete chromosomal replication but do not initiate new rounds and arrest with an integer number of chromosomes. The number of chromosomes corresponds to the growth rate prior to arrest. In polyploid arrested cells, the chromosomal regions at which replication initiates are segregated, whereas the termini regions remain colocalized. The *E. coli* chromosome remains decondensed and unsegregated during arrest and rapidly resumes replication and segregation, concomitant with chromosome condensation, upon release. The protein SeqA, a DNA binding protein and negative regulator of replication, is necessary for enforcing this arrest.

## Introduction

Bacterial cells encounter varied environmental stresses and make appropriate adjustments to ensure survival. One such stress is amino acid starvation, which triggers physiological reprogramming known as the “stringent response”. Signaling of the stringent response is achieved by accumulation of effector nucleotides, guanosine tetra- and pentaphosphate ppGpp and pppGpp (reviewed in [1]), with the former the predominant and more stable of the two species. Two enzymes, RelA and SpoT, control the levels of ppGpp: RelA (PSI, or ppGpp synthetase I) synthesizes ppGpp in response to uncharged tRNA, the consequence of amino acid starvation; SpoT (PSII) possesses weak synthetase activity and is the sole hydrolase for ppGpp degradation.

The stringent response is RelA-dependent and is the most well-studied of stress responses signaled by ppGpp (reviewed in [1]). RelA associates with the ribosome and is triggered to produce ppGpp when the ribosome stalls with an uncharged tRNA in the acceptor site. This causes a dramatic alteration in gene expression, including the reduction of rRNA synthesis and increased transcription of amino acid biosynthesis genes [2]. These changes result from direct binding of ppGpp to RNA polymerase, aided by the transcription factor, DksA [3],[4]. Although it has been reported that ppGpp accumulation promotes arrest of replication, in addition to the well-studied transcriptional changes, the mechanism of the cell cycle arrest remains unclear [5],[6] and is the subject of our investigation.

The cell cycle of *E. coli* under low nutrient conditions is similar to that of eukaryotic cells, with a period prior to initiation of replication (“B period”, equivalent to eukaryotic G1), a period with ongoing DNA synthesis (“C period”, equivalent to S phase) and a period after completion of replication but before cell division (“D period”, equivalent to eukaryotic G2 phase). In medium rich in nutrients, *E. coli* cell cycle is accelerated such that it becomes faster than the time necessary to replicate the entire chromosome; under this circumstance, replication cycles overlap and cells are born with partially replicated chromosomes, whose replication was initiated in its mother or even grandmother [7].

In *E. coli*, replication initiates at the *oriC* locus and proceeds bidirectionally to completion within the terminator region, *ter* [8]. Replication initiation is tightly controlled by the AAA+ ATPase, DnaA [9]. Binding to *oriC* followed by loading of the DnaB helicase depends on levels of DnaA-ATP [9], correlated with achievement of a critical cell mass [10]–[12]. Firing of sister origins occurs in synchrony, such that cells contain 1, 2, 4, 8, 16, etc. copies of the *oriC* locus.

“Sequestration”, the binding of SeqA to a hemimethylated origin is a component of initiation control [13]–[15]. SeqA binds newly replicated, hemimethylated GATC sequences throughout the entire genome [16],[17]. Within *oriC*, SeqA binding prolongs its hemimethylated status and blocks DnaA-origin interaction until the region becomes fully methylated by Dam methyltransferase [13],[18],[19]. This contributes to the “eclipse period”, the time within a cell cycle when reinitiation is actively prevented [20], which is defective in mutants lacking SeqA and Dam [15], [21]–[23].

In addition, SeqA is implicated in chromosome segregation and nucleoid organization [24],[25]. In the absence of SeqA, DNA becomes more negatively supercoiled and nucleoids appear more decondensed [15],[26],[27]. Immunofluorescence microscopy of SeqA and GFP-tagged SeqA reveals foci colocalized with the replisome, consistent with SeqA binding to hemimethylated DNA as it emerges from the replication fork [24], [25], [28]–[30]. Under conditions of fast growth with overlapping replication cycles, SeqA promotes the colocalization of sister origins in *E. coli*, in a manner independent of sequestration at *oriC* [31]. SeqA overexpression also interferes with chromosome segregation [32].

It has been reported that stringent response in *E. coli* promotes replication arrest via inhibition of initiation [33]–[36]. Phase and fluorescent microscopy show that cells experience a reduction in cell size and contain a single nucleoid at mid-cell after ppGpp accumulation [34]. Initial reports showed limited replication at *oriC* after stringent onset [36] and later studies revealed that cells have a significant reduction, though not a complete block, in septum formation and cell division [34]. We reexamine replication arrest induced by the stringent response using flow cytometric techniques and implicate SeqA as a contributor to this arrest.

Our studies reveal that under the stringent response, cells appear to complete replication and arrest at an integer DNA content corresponding to their pre-arrest growth conditions. This arrest appears to be dependent on RelA-dependent ppGpp synthesis. Stringent cells always contain one decondensed nucleoid; in *relA* mutants that fail to arrest, nucleoid compaction is apparent, similar to that seen upon treatment of cells with the translational inhibitor, chloramphenicol. Although marker frequency analysis shows that the ratio of *oriC* to *ter* is 1 after stringent arrest, visualization of *oriC* and *ter* by ParB-GFP binding shows that sister loci at the termini remain colocalized, whereas the *oriC* loci have separated. Stringent cell cycle arrest is dependent on SeqA and Dam, in a manner that is only partly dependent on GATC sites near the origin.

## Results

### Stringent Cell Cycle Arrest in Wild-Type Cells under Different Growth Conditions

To determine the extent of DNA replication in populations of *Escherichia coli* K12 cells growing in defined media, we used flow cytometry for DNA content using the stain PicoGreen (Invitrogen). We examined DNA content in cells at different growth rates, with and without treatment with serine hydroxamate, a derivative of serine that acts as a competitive inhibitor of seryl-tRNA synthetase, eliciting the stringent response [37]. This was compared to cells incubated with rifampicin and cephalexin, so-called “run-out” conditions that are used routinely to judge cell cycle status of *E. coli*. These antibiotics block initiation of replication and cell division, respectively; under this regimen, ongoing replication forks are completed and cells arrest in the D period. Under run-out conditions, the DNA content reflects the number of *oriC* loci in the cell at the time of treatment.

**Figure 1:**
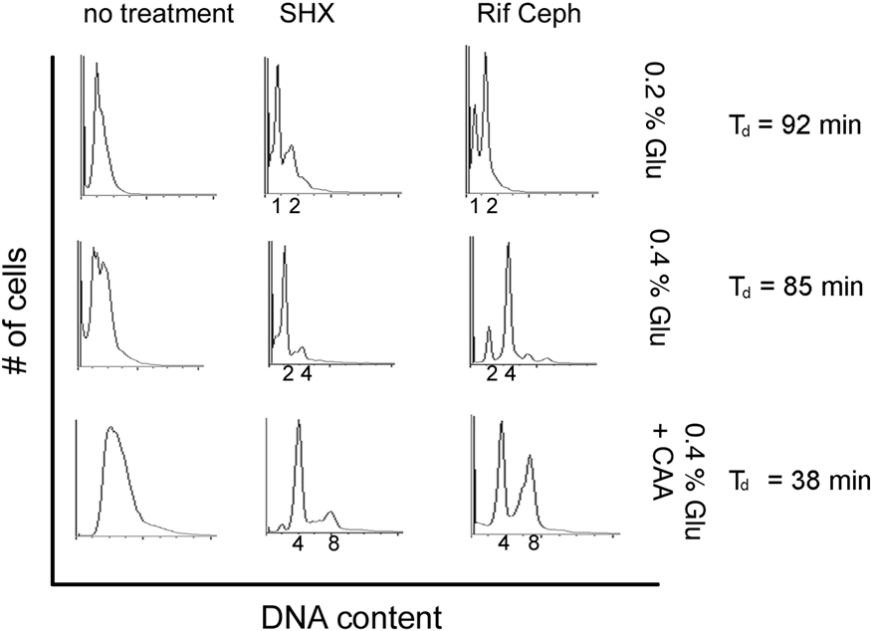
Flow cytometric analysis of cell cycle and effects of the stringent response. Cells were grown in 0.2% glucose, 0.4% glucose, or 0.4% glucose 0.2% casamino acids—M9 minimal media. DNA content for wild type and mutant cells before treatment (left panel), treated with serine hydroxamate (SHX) for 1.5 h (middle panel), or rifampicin or cephalexin for 4 h (right panel). Samples were taken for determination of DNA content by PicoGreen fluorescence (see Materials and Methods). The fluorescent scale (x-axis) corresponds to chromosome equivalents as indicated. The population doubling times, T_d_, for each growth condition are indicated.

With no treatment, there is a broad distribution of cellular DNA content, indicative of DNA replication, asynchronous in the population (Figure 1). After treatment with rifampicin and cephalexin, replication is completed and cells assume an integer DNA content reflective of the growth rate. In low glucose medium, the majority of cells appear to be replicating and arrest at 2N DNA content after run-out; a subpopulation appears not to have initiated DNA replication at the time of rifampicin and cephalexin treatment and arrest at 1N. In higher glucose minimal medium, the cell cycle is accelerated such all cells appear to be replicating. Some arrest after run-out as 2N but a proportion of cells have initiated a second round of replication prior to completion of the first and arrest at 4N. In high glucose with casamino acids (CAA), all cells have initiated a second round, some with a third round, and arrest after run-out as a mixture of 4N and 8N cells.

In each growth medium, treatment with serine hydroxamate to induce the stringent response for 1.5 hr caused the population to assume an integer DNA content, similar to treatment with rifampicin and cephalexin, indicating an inhibition of cell cycle progression and DNA replication. In each case, the integer DNA content at stringent arrest was somewhat lower than that seen with rifampicin and cephalexin treatment. This indicates that some stringent arrested cells undergo cell division after cessation of replication. Although some stringent cells may arrest in D period (after completion of replication and prior to division, G2-like) as do rifampicin and cephalexin-treated cells, there is a preference for stringent arrest in B period (after division but prior to replication re-initiation, G1-like).

### RelA-Dependent ppGpp Synthesis Is Necessary and Sufficient for Cell Cycle Arrest

**Figure 2:**
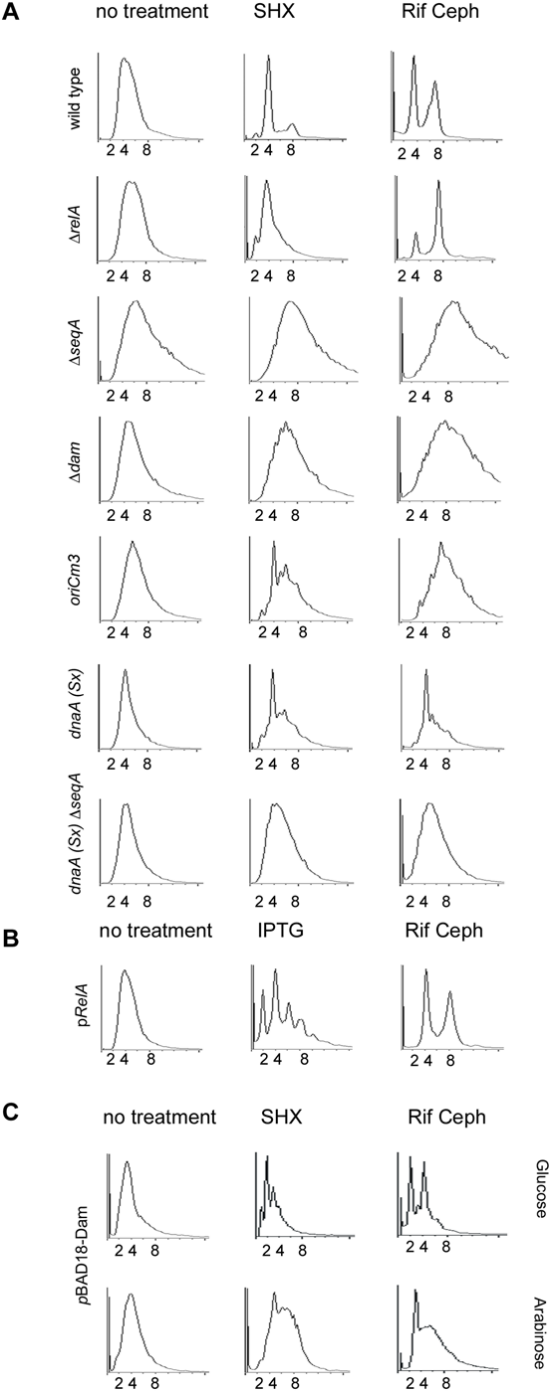
Stringent arrest depends on RelA, SeqA, and DNA hemi-methylation. (A–C) Flow-cytometric analyses of PicoGreen stained cells growing in M9 CAA media. Samples were prepared for determination of DNA content by PicoGreen fluorescence as in Figure 1. (A) Wild-type and mutant cells before treatment (left panel), treated with serine hydroxamate (SHX) for 1.5 h (middle panel), or rifampicin or cephalexin for 4 h (right panel). (B) DNA content for wild type cells harboring the pRelA′ plasmid before (left panel) and after induction with IPTG (middle panel), or rifampicin and cephalexin for 4 h (right panel). (C) Wild-type cells harboring pBAD18-Dam were grown in M9 glucose CAA to repress expression (top row) or M9 arabinose CAA to induce expression (bottom row). Left and center panels show before and after treatment with serine hydroxamate (SHX) for 1.5 h, respectively. Right panels show treatment with rifampicin and cephalexin for 4 h. Cultures were grown at 37°, except for strains containing *dnaA*(Sx), which were grown at 34°.

Under the fastest growth condition, we examined *relA* mutants, defective in ppGpp production elicited by serine hydroxamate. Mutants in *relA* were noticeably impaired for arrest after serine hydroxamate treatment and the cellular DNA content of the population was broadly distributed, indicative of ongoing replication (Figure 2A). In the presence of rifampicin and cephalexin, *relA* mutants assumed integer DNA content similar to wild-type cells, but with slightly more 8N relative to 4 N peaks. This finding confirms our expectation that ppGpp production by RelA is required for cell cycle arrest during the stringent response.

We also examined wild-type cells in which the stringent response was induced by high levels of a catalytically active truncated RelA fragment, RelA′. Overexpression of RelA′ causes ppGpp production, independent of idle ribosomes [38]. This method has the advantage of ppGpp accumulation independent of ribosome signaling and which is not accompanied by translational stalling. This allows us to distinguish ppGpp-specific effects from those associated indirectly with starvation or translation inhibition. If ppGpp accumulation is sufficient to induce cell cycle arrest, we would expect DNA profiles from pRelA′ induced cells to resemble those after treatment with serine hydroxamate. Indeed, cell cycle arrest and integer chromosome number was apparent by flow cytometry in IPTG-induced pRelA′ cells as compared to non-induced cells (Figure 2B). This confirms that accumulation of ppGpp, rather than inhibition of translation or starvation itself, is responsible for the arrest. We did note that the integer chromosome number after RelA′ overexpression was somewhat lower, with a more predominant 2N peak, and more asynchronous, with a strong 6N peak, than that seen for serine hydroxamate. This may be a consequence of higher ppGpp levels induced by RelA′ overexpression.

### Stringent Arrest Requires SeqA and Dam Methylase

Because of the implication of the DNA binding protein SeqA in control of both chromosome initiation and segregation, we tested Δ*seqA* mutants for DNA content after induction of the stringent response. We found them to be virtually blind to serine hydroxamate treatment (Figure 2A) and DNA profiles resemble untreated cells. As has been noted previously [15],[39],[40], *seqA* mutants are also unable to assume integer DNA content after run-out with rifampicin and cephalexin, potentially because initiation is no longer sensitive to rifampicin or because ongoing forks fail to proceed to completion. The former explanation is suggested by the fact that the mean DNA content per cell in the *seqA* mutant cell population appears to increase substantially even after rifampicin/cephalexin treatment, as compared to untreated cells.

SeqA binds preferentially to GATC sites hemi-methylated by Dam (DNA adenine methylase) over unmethylated sites [20] and therefore we also tested *dam* mutants, which are expected to resemble *seqA* defective strains. We observed strains lacking Dam methylase showed a stringent cell cycle arrest defect similar to Δ*seqA* strains (Figure 2A). As *seqA*, *dam* mutants show no “run-out” after treatment with rifampicin and cephalexin (Figure 2A), although average cellular DNA content increases, suggesting rifampicin-resistant replication.

Because of SeqA's preference for hemi-methylated sites, Dam-overproducing cells also perturb SeqA binding to GATC sequences and cause defects in initiation control. Overproduction of Dam causes rapid conversion of hemimethylated DNA to fully methylated DNA, decreasing SeqA's ability to bind to these sites [13]. We would therefore expect Dam-overexpressing cells to exhibit defects in stringent cell cycle arrest, similar to *seqA* and *dam* mutants. To test this, we engineered otherwise wild-type cells to overexpress Dam from an arabinose-inducible promoter. This strain exhibited cell cycle arrest after treatment with serine hydroxamate when grown in the presence of glucose but failed to shift DNA content fully to an integer amount when Dam was induced by arabinose (Figure 2C). There was, however, evidence of a slight 2N peak in Dam-induced wild-type cells: this may represent a subpopulation that arrests replication because Dam levels are not sufficiently high or because they have lost the plasmid. After treatment with rifampicin and cephalexin, glucose-grown cells were primarily 2N and 4N whereas many arabinose-grown, Dam-overexpressing cells failed to assume integer DNA content. As *seqA* and *dam* mutants, Dam-overexpressing cells may exhibit rifampicin-resistant replication initiation that prevents “run-out” to integer DNA content.

DNA content is notably higher in *seqA* mutant strains due to loss of initiation restraint and subsequent over-replication [15]. To test whether the tendency to overinitiate obscured a functional stringent arrest in Δ*seqA* cells, we used a genetic suppressor of *seqA* in *dnaA*. The hypomorphic *dnaA*(Sx) allele lowers the frequency of initiation at cold-temperatures and suppresses the overinitiation property of Δ*seqA* mutants at its permissive temperature [40],[41]. We confirmed that *dnaA*(Sx) itself does not influence stringent cell cycle arrest. *dnaA*(Sx) cells displayed a stringent arrest after treatment with serine hydroxamate as indicated by a shift to an integer number of chromosomes, similar to run-out conditions (Figure 2A). When combined with the *dnaA*(Sx) allele, Δ*seqA* cells maintain a DNA content similar to wild-type cells in the absence of any treatment (Figure 2A). The *dnaA*(Sx) Δ*seqA* mutant did not successfully arrest after treatment with serine hydroxamate, although DNA content remained within the range that was comparable to arrested wild-type cells. This suggests that failed stringent arrest in Δ*seqA* cells is due to a lack of SeqA function and is not an indirect consequence of hyper-initiation or excessive DNA content.

### The Role of *oriC* in Stringent Arrest

SeqA appears to have two distinct functions: it binds to hemimethylated DNA sites at the origin and, somewhat more transiently, to newly replicated DNA throughout the chromosome. To distinguish between the origin and other sites of SeqA binding, we examined DNA content after stringent arrest in an *oriC*m3 strain, which is specifically defective in *oriC* sequestration. *oriC*m3 strains have eight of the GATC sequences within *oriC* converted to GTTC, causing a decreased affinity for SeqA, asynchronous replication, shortened eclipse periods and asymmetric cell division, equivalent to strains lacking SeqA altogether [39]. These mutations should not affect SeqA binding to hemi-methylated DNA revealed after replication of other regions of the chromosome. Flow cytometry of *oriCm3* cells shows a modest increase in DNA content, presumably due to hyperinitiation caused by defective origin sequestration. After treatment with serine hydroxamate, the DNA content indicates that stringent arrest is partially intact in the *oriC*m*3* strain, with a prominent 4N arrest peak. This suggests that there is a critical role of SeqA in enforcing the stringent response by interactions to DNA sites outside the origin. Mutants in *oriC*m*3*, like those in *dam* and *seqA*, showed poor run-out to integer chromosomal content after treatment with rifampicin and cephalexin.

### Segregation of Specific Chromosome Regions after Stringent Arrest

To examine segregation patterns of specific locations of the chromosome, we used strains that contain a *parS* sequence inserted adjacent to the origin or to the terminus. The *parS* sequence provides a binding site for the plasmid-expressed fusion protein, GFP-ParB, to allow visualization of the locus of interest [42]. To enhance GFP fluorescence, these experiments were performed at lower temperature, 34°, and flow cytometry was performed on these cultures in parallel to discern their cell cycle status.

**Figure 3:**
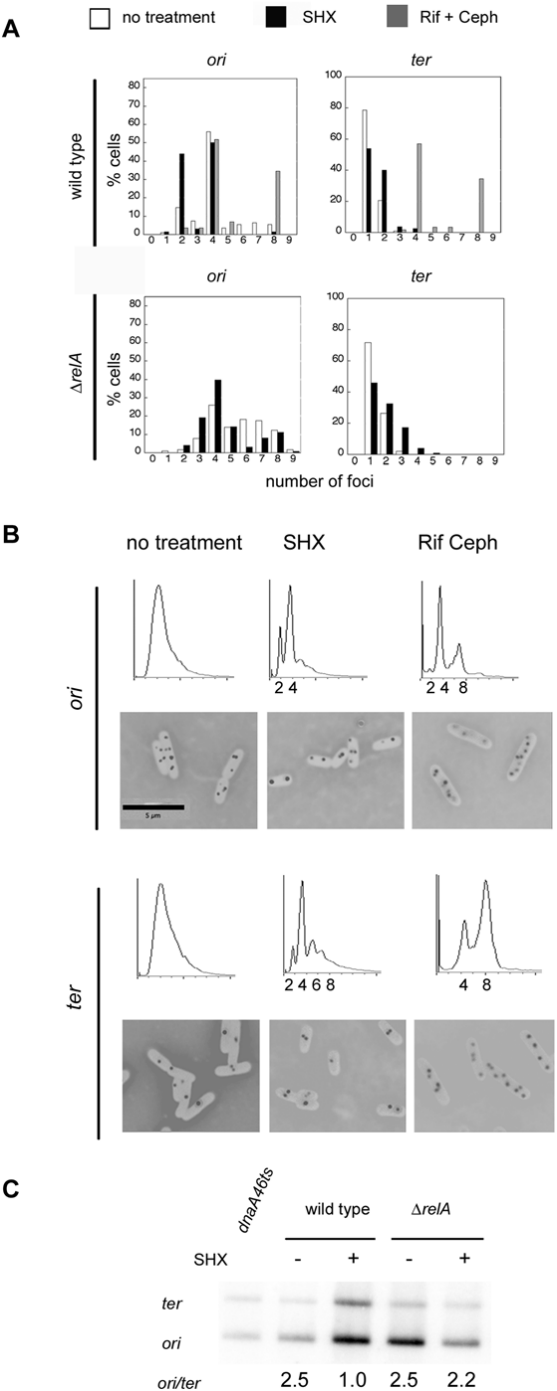
Origin and termini number and localization in wild type and mutant cells. Strains with GFP marked- *ori* (84′) and *ter* (33′) regions were visualized using the GFP-ParB/*parS* system. For optimal GFP signal, cells were grown at 34°C. (A) Histograms of foci per cell in wild type and Δ*relA* cells growing in M9 glucose CAA medium. The number of foci was determined by counting, and number of chromosomes was determined by corresponding flow cytometry histograms shown in Figure 3B. Foci counts were determined before (white bars) and after treatment with serine hydroxamate (black bars) or rifampicin and cephalexin (grey bars). (B) Flow-cytometric analysis and corresponding fluorescent images of wild-type cells from Figure 3A shown before (left panel) and after treatment with serine hydroxamate (middle panel), or rifampicin and cephalexin (right panel). (C) Ratio *ori* to *ter* ratio by Southern blotting of chromosomal DNA from wild-type strains and various mutants grown at 37°. Cultures were treated with and without serine hydroxamate. Chromosomal DNA was extracted and digested (see Materials and Methods). Probes were to the chromosomal regions proximal to *ori*, (*gidA*, 84.3 min) and *ter* (*relB* 34.8 min).

For growth in minimal medium plus casamino acids at 34°, examination of *oriC* and *ter* foci via ParB-GFP showed that stringently-arrested wild-type cells have two or four distinct *oriC* foci (Figure 3A and 3B). However, the number of visible *ter* foci was one or two, half that seen for *oriC* and similar to that seen for untreated cells. Marker frequency analysis by quantitative Southern blots for *oriC* and *ter* showed that with no treatment, the *oriC* to *ter* ratio was 2.5 (Figure 3C), indicating that replication was ongoing. After induction of the stringent response, the *oriC/ter* ratio in wild-type cells was 1.0 (Figure 3C), indicating that replication was complete. In contrast, *relA* mutant cells retained an *oriC/ter* ratio of greater than 2, even after serine hydroxamate treatment (Figure 3C). In similar analysis, the *oriC/ter* ratio of *dnaA*(Sx) *seqA* mutant cells was 2.8 before treatment and 2.7 after serine hydroxamate, confirming that replication is ongoing after induction of the stringent response in these strains.

The 2∶1 ratio of *oriC* to *ter* foci after serine hydroxamate treatment of wild-type cells, despite the fact that the chromosome appears to have completely duplicated from the marker frequency analysis, suggests that *ter* loci remain colocalized after replication. In contrast, colocalization of *ter* was not observed in wild-type cells arrested in the cell cycle by rifampicin and cephalexin: under these conditions the number of *ter* foci are equivalent to those seen for *oriC* (Figure 3A and 3B). This confirms that colocalization of *ter* seen during the stringent response is not likely to be a simple artifact of the ParB-GFP visualization system. Rather, there appears to be a distinct chromosome organization pattern enforced by the stringent response, and it is unlike that seen with other types of cell cycle inhibitors.

### Stringent Arrest in Wild-Type Cells Occurs with 1 Single, Decondensed Nucleoid

**Figure 4:**
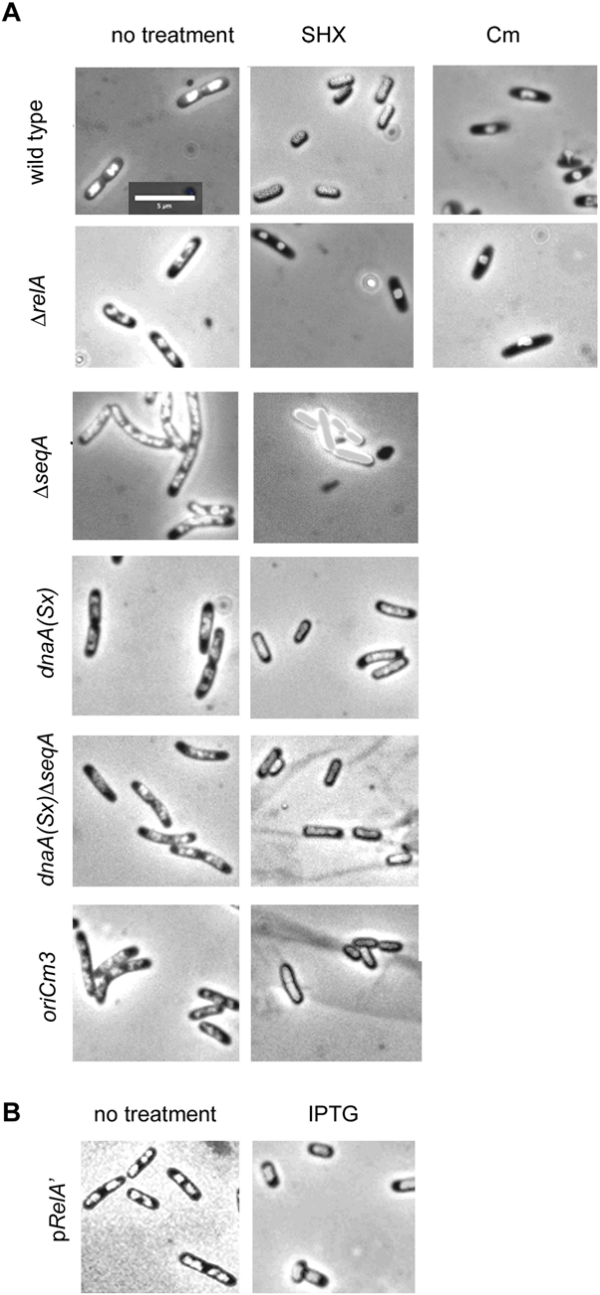
Nucleoids of stringent cells. Cells stained with DAPI from cultures taken before and after induction of the stringent response. Cells were grown in M9 glucose CAA medium, fixed, and stained as described in Materials and Methods. (A) Wild type and mutant cells before (left panel) and after treatment with serine hydroxamate for 1.5 h (middle panel), and, in the case of wild type and Δ*relA* cells, 300 µg/ml chloramphenicol for 1.5 h (right panel). (B) MG1655 cells harboring pRelA′ before (left panel) and after (right panel) induction with IPTG.

We examined the nucleoid morphology of stringent cells by DAPI-staining and fluorescence microscopy. Untreated wild-type cells growing in minimal CAA medium showed 1 or 2 nucleoids and had apparent signs of division and chromosome segregation (Figure 4A, left panel). DNA-free zones of the cytoplasm were apparent. After treatment with serine hydroxamate, signs of cell division were absent and all cells contained one nucleoid (Figure 4A, middle panel). The nucleoids appear decondensed, filling nearly the entire cell volume. This was quite different from cells treated with the translation inhibitor chloramphenicol, with a single, very condensed nucleoid structure at midcell (Figure 4A, right panel), consistent with previous reports [43]. From the observation of chloramphenicol-collapsed nucleoids, it has been argued that translation and anchoring of membrane proteins is required to extend the nucleoid throughout the cytoplasm. Despite a potential down-regulation of translation induced by serine hydroxamate, the nucleoid remained surprisingly decondensed, even more so than untreated cells. The decondensed nucleoid was not just a consequence of serine hydroxamate treatment: we saw similar appearance in cells in which the stringent response had been induced by pRelA′ overexpression (Figure 4B).

We also examined nucleoid morphology in Δ*relA* strains (Figure 4A). Untreated Δ*relA* nucleoids appear similar to wild-type; however, when treated with serine hydroxamate, nucleoids in Δ*relA* cells have a condensed appearance, similar to cells treated with chloramphenicol. This may indicate that the absence of capacity to synthesize ppGpp causes a collapse in translational capacity upon serine hydroxamate treatment, reflected in nucleoid appearance. Or, it could mean that ppGpp induces some active nucleoid decondensation process that is absent in *relA* strains. Interestingly, mutants in *seqA*, despite their failure to arrest replication, appeared to retain the decondensed nucleoid character of wild-type cells after serine hydroxamate treatment. Although this is difficult to see in *seqA* strains because of their excessive DNA content, nucleoid decondensation after serine hydroxamate is also apparent in *dnaA*(Sx) *seqA* strains that have more normal DNA content per cell. This may indicate that *seqA* affects only a subset of the responses to ppGpp (see below). Mutants in *oriC*m*3* also exhibited nucleoid decondensation similar to wild-type cells after serine hydroxamate treatment.

### Release from Stringent Arrest

**Figure 5:**
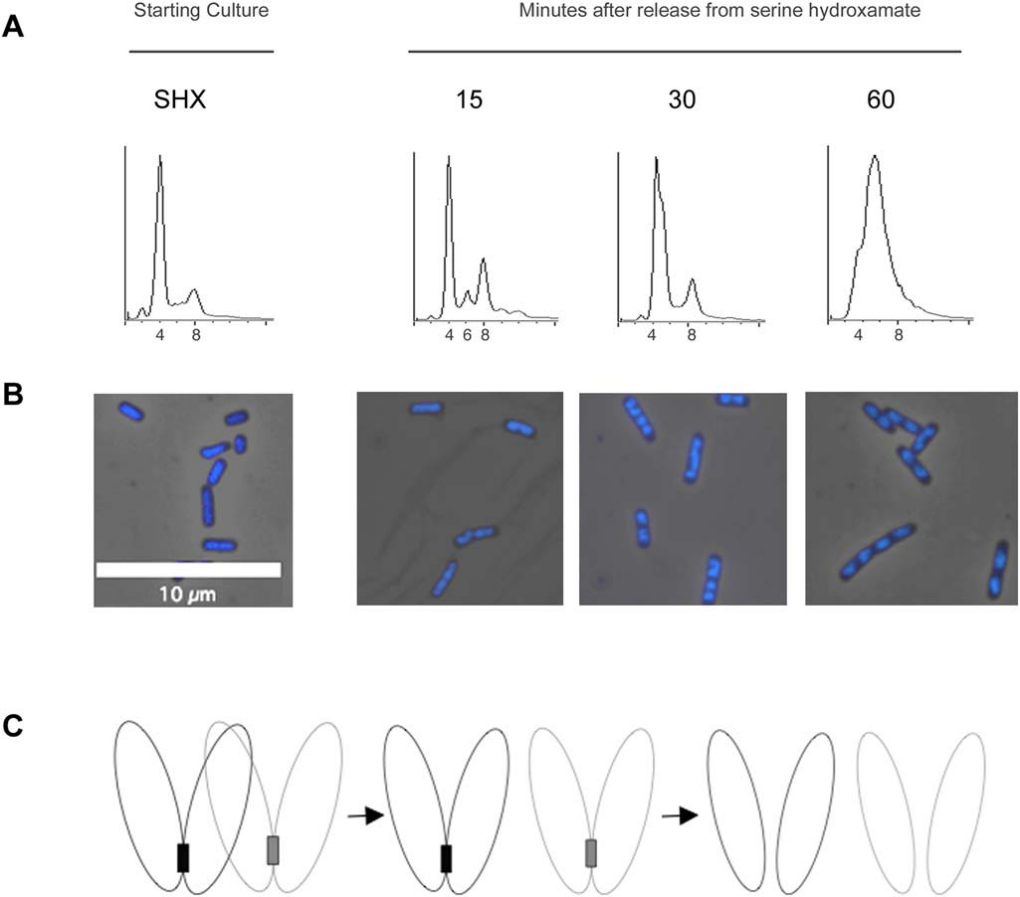
Release from stringent arrest. Wild type cells in M9 Glucose CAA medium were treated with serine hydroxamate for 1.5 h as previously described. Cells were then washed of serine hydroxamate, incubated in fresh medium, and samples were taken for flow cytometric analysis for DNA content and DAPI staining as previously described. (A) DNA content histograms and (B) corresponding fluorescent DAPI images of cultures incubated with serine hydroxamate for 1.5 h and of cultures at 15, 30, and 60 min after being released from serine hydroxamate. (C) Model for chromosome segregation patterns for 4N cells released from stringent arrest. Cohesion of sister chromosomes help enforce the sequence of segregation of chromosomes from the single nucleoid mass. Pairs of sister chromosomes held in cohesion at their termini segregate first at midcell. After release of cohesion, sisters separate at ¼ positions.

We wondered how cell cycle patterns would be reset after release from arrest. Would cells resume replication in a pattern similar to that prior to arrest? Or would cells be obligated to segregate each chromosome, divide and initiate replication from 1N progeny? To examine this, after 90 minutes of stringent arrest induced by serine hydroxamate, wild-type cells in minimal M9 Glucose CAA medium were washed and allowed to resume growth. DNA content was followed over time by flow cytometry and nucleoid appearance was monitored by DAPI staining (Figure 5A and 5B). As early as 15 minutes after release, some signs of nucleoid segregation were apparent. Segregation of two nucleoids appeared first at midcell; later segregation at the ¼ positions to form 4 nucleoids was detected. We think this reflects sequential and ordered segregation of chromosomes: sister-chromosome pairs held in cohesion segregate first, followed by separation of sisters (see schematic in Figure 5C). From 15 to 30 minutes, DNA content in the entire population appeared to increase, concomitant with nucleoid condensation and segregation. Therefore, cells appear to assume DNA content equivalent to conditions before the arrest, suggesting that replication patterns are reset quickly upon release.

### Inhibition of Colony Formation by ppGpp Accumulation Is Relieved in Δ*dam* and Δ*seqA* Mutants

Growth is inhibited in wild-type *Escherichia coli* cells with elevated ppGpp levels and they are unable to form colonies [44]. We wondered whether this is a consequence of cell cycle arrest induced by the stringent response and, moreover, if the stringent cell cycle arrest defects in Δ*dam* and Δ*seqA* would permit colony formation during chronic ppGpp production. Wild type, Δ*seqA*, Δ*dam*, and *oriC*m3 cells containing the IPTG-inducible pALS13 plasmid producing truncated RelA′, were grown on M9 minimal CAA ampicillin plates containing various concentrations of IPTG. A control plasmid, pALS14, contains a truncated 1.2 kb fragment of RelA that has no enzymatic activity [38]. Colony formation in wild-type cells was severely inhibited by induction of RelA′ (Table 1) but not with the nonfunctional RelA- protein. Similar inhibition of plating was obtained by induction of pALS10, expressing wild-type RelA^+^ (data not shown). Δ*seqA* and Δ*dam* cells, however, maintained the ability to form some colonies in the presence of elevated ppGpp concentrations, with plating efficiencies dramatically elevated, over 1000-fold, in the presence of IPTG (Table 1, and data not shown). On the other hand, *oriC*m*3* cells had only a modestly increased plating efficiency of about 10-fold. This is consistent with our flow cytometric results and suggests that an origin compromised for SeqA interaction has only a partial defect in the stringent cell cycle arrest, considerably less severe than strains lacking all SeqA function or Dam methylase. Cells that successfully formed colonies under stringent response induction in Δ*seqA* or Δ*dam* genetic backgrounds were no more resistant than the original population upon rechallenge with IPTG (data not shown), indicating that no heritable change arising during growth, which is a special concern for *dam* mutator strains, had allowed them to escape preferentially.

**Table 1:** Efficiency of plating with chronic stringent response induction.

Genotype	pALS13	pALS14
	pRelA′	pRelA−
wild type	1.6±0.2×10^−6^	1.0±0.6
Δ*seqA*	5.2±2.3×10^−3^	1.2±0.004
Δ*dam*	7.8±2.7×10^−3^	0.50±0.22
*oriCm3*	2.4±1.2×10^−5^	0.94±1.5

Plating efficiency of replication mutants harboring pALS13 (RelA′) or pALS14 (RelA^−^) after induction with 100 µM IPTG.

The inability of cells to form colonies under constitutive ppGpp accumulation appears to be, in part, a consequence of cell cycle arrest. We do note that the rescue of plating by *seqA* and *dam* is not 100%, indicating that transcriptional reprogramming, translational inhibition or other aspects of the stringent response, which remain intact in *seqA* and *dam* mutants (see below), may also be inhibitory to growth and division.

### Δ*seqA* and Δ*dam* Have Normal Stringent Control in Other Respects

**Figure 6:**
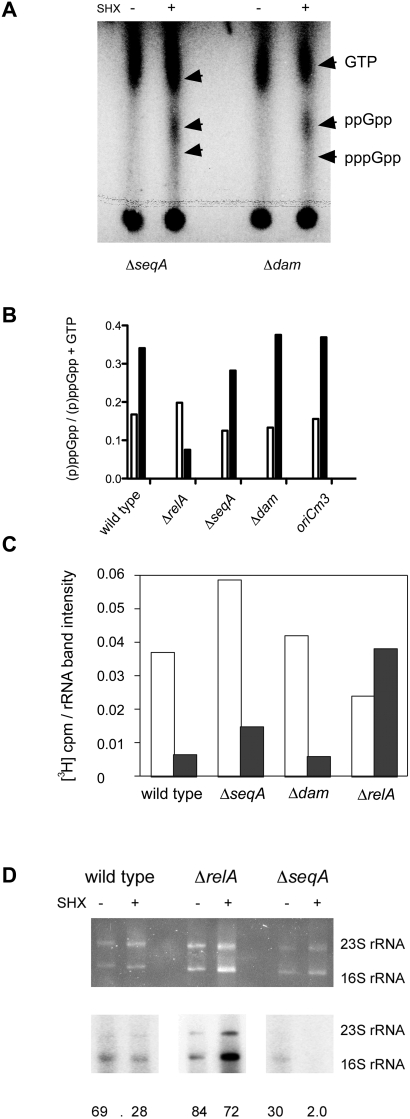
ppGpp production and stringent effects on rRNA labeling are normal in Δ*seqA* and Δ*dam* strains. (A) Autoradiograms of polyethyleneimine thin-layer chromatography plates showing detection of ppGpp in *seqA* and *dam* cells. Cells were grown in low-phosphate medium and uniformly ^32^P_i_-labeled with 100 µCi/ml before treatment with SHX for 10 min. (B) (p)ppGpp levels normalized according to GTP levels before and after 10 min of incubation with SHX. Open bars indicate levels before treatment (t = 0) and solid bars after treatment (t = 10). (C) Incorporation of [^3^H]-uridine in pulse-labeled cultures treated with and without serine hydroxamate. Cultures were treated with serine hydroxamate (SHX) at t = 0. Addition of [^3^H] uridine occurred at 15 min, followed by a chase with excess cold uracil at 25 min. Cultures were isolated and RNA extracted at 30 min. RNA was purified and separated by electrophoresis; incorporation of radioactive nucleotide was determined by extracting and dissolving rRNA bands and measuring cpm by scintillation counting. Normalization of counts was achieved by dividing by the cpm of [^3^H] over the amount of total rRNA, as indicated by ethidium bromide fluorescence band intensity. Cultures not treated with serine hydroxamate are open bars, and cultures treated with serine hydroxamate are solid bars. (D) Incorporation of ^32^P_i_ into rRNA in cultures treated with and without SHX. Cultures were grown in low-phosphate medium and treated with SHX at t = 0 followed by addition of 200 µCi of [^32^P]H_3_PO_4_ at t = 15 min. Total RNA was isolated at t = 30 min. Top panel: UV-light induced fluorescence image of rRNA on a native agarose gel stained with ethidium bromide. Middle panel: autoradiogram of rRNA from top panel after transfer to a nylon membrane. Bottom panel: ratio of radiolabeled rRNA to the amount of UV-light–induced fluorescent rRNA.

One possible explanation for the inability of Δ*dam* and Δ*seqA* strains to arrest cell cycle during the stringent response is that production of ppGpp, or its ability to interact with RNA polymerase, for some reason, is defective. To investigate this, we examined ppGpp production directly by ^32^P-phosphate labeling and separation of nucleotides by thin-layer chromatography (Figure 6A and 6B). The more slowly-migrating form corresponding to ppGpp was apparent in wild-type, *seqA*, *dam* and *oriCm3* strains after treatment with serine hydroxamate; this was not seen for *relA* strains.

Another characteristic of stringent control in wild type cells is the reduction of stable RNA synthesis [44]. Uridine uptake is also reduced by induction of the stringent response [45]. By pulsing cells with [^3^H]-uridine, treated with or without serine hydroxamate, we examined the amount of newly labeled rRNA synthesis over time (Figure 6C). Wild-type cells treated with serine hydroxamate display a decrease in labeled rRNA compared to untreated cultures. Δ*relA* cells, defective in stringent control, maintain labeled rRNA production at high levels, blind to treatment with serine hydroxamate. Like wild-type, Δ*seqA* and Δ*dam* cells showed reduced labeled rRNA after serine hydroxamate addition.

Because the latter experiment reflects both down-regulation of rRNA synthesis, as well as reduction in uridine uptake, we also examined pulse-labeling of rRNA with inorganic phosphate, relative to total levels as determined by ethidium bromide staining, which should be more reflective of rRNA synthesis. Labeling of rRNA decreased over 2-fold after treatment with serine hydroxamate in wild-type cells and 15-fold in Δ*seqA* mutant cells; incorporation of ^32^P into rRNA was not strongly affected by serine hydroxamate treatment in *relA* mutants that fail to synthesize ppGpp. These experiments suggest that *seqA* and *dam* mutants have no defect in production of ppGpp or other aspects of the stringent response, including transcriptional down-regulation of rRNA synthesis and possibly uridine uptake, and therefore are specifically impaired in cell cycle response to ppGpp.

## Discussion

In this study, we have characterized the replication arrest that occurs in *Escherichia coli* cells under the stringent response. We confirm a previous report that initiation but not elongation of replication is blocked and that RelA-dependent ppGpp synthesis is required for full arrest [34]. The majority of cells appear to arrest in the B period, prior to initiation, and contain an integer number of chromosomes appropriate for the growth medium prior to arrest. A minority of cells may arrest in the D period, after replication is completed but prior to chromosome segregation. In either case, stringent *E. coli* cells have unsegregated, but apparently fully-replicated, chromosomes, indicating a new cell cycle point of stringent control.

We have identified mutants, Δ*seqA* and Δ*dam*, that cannot control replication in response to ppGpp and continue to divide in the presence of chronic stringent induction that is sufficient to inhibit growth of wild-type strains. Overproduction of Dam methylase is also sufficient to uncouple the control of cell cycle from the stringent response, suggesting that stringent cell cycle arrest is regulated by SeqA interactions with hemi-methylated GATC sites. Other aspects of the stringent response, including down-regulation of rRNA synthesis, decondensation of nucleoids, and a diminution in cell size (data not shown), appeared to be intact in *seqA* mutants. This implies that SeqA and Dam specifically control cell cycle and do not have global effects on the stringent response, including levels of ppGpp.

In bacteria, as well as other organisms, control of the cell cycle is likely to be an important component of stress responses. Our previous work suggested that SeqA may limit replication in the face of chronic DNA damage [40]. In the case of the stringent response, it may be advantageous to limit replication in the presence of diminishing translational capacity since stalled forks may be subject to cleavage and collapse, causing the demise of both chromosomes. Arrest with two or more replicated chromosomes provides an opportunity for recombinational repair if one of the chromosomes becomes damaged and two potential templates for gene expresssion. Decondensation of the nucleoid, potentially induced by the stringent response, may facilitate the dynamic interactions between sister chromosomes that are required for recombinational repair.

Bacterial cells fail to proliferate when ppGpp levels rise, such as caused by overexpression of RelA or loss of the ppGpp hydolase SpoT (reviewed in [1])–our experiments suggest that this is due in large part to SeqA-dependent arrest of cell cycle. Mutants in *dam* or *seqA* are dramatically relieved of growth inhibition by overexpression of RelA and are able to form colonies, albeit with some loss of plating efficiency, likely the result of down-regulation of translational capacity [46].

### Stringent Cell Cycle Arrest Points

Previous results suggested that initiation of replication is blocked when ppGpp levels are high and that elongation of replication continues to completion after ppGpp accumulation [34]–[36]. Our findings confirm this and suggest that chromosome segregation is an additional point of stringent regulation. Stringent cells arrest with unsegregated nucleoids with colocalized termini. This is consistent with reports showing that cell division, though not completely inhibited, does not likely proceed until each cell has 1 chromosome [34].

Stringent cell cycle arrest points differ between *Escherichia coli* and *Bacillus subtilis*. *B. subtilis* arrests replication elongation by ppGpp inhibition of primase activity [47]. It is unclear why *E. coli* does not perform this C-period (“intra-S phase”-like) arrest, although it remains possible that elongation is slower in stringent cells or punctuated with stalling events. We also do not know the threshold levels of ppGpp or starvation that elicit replication arrest in the two organisms. It remains possible that elongation arrest in *E. coli* could occur under more stringent conditions or occurs in a subpopulation of cells, obscured by arrest in B and D periods. During the stringent response in *E. coli*, GTP levels do not fall more than 50% [46]; in contrast, *Bacillus subtilis* experiences a drop in GTP concomitant with ppGpp accumulation and its stringent response may be an indirect consequence of depletion of GTP pools [48]. For this reason, *E. coli* may respond more sensitively to ppGpp, enabling it to complete replication before collapse of nucleotide pools that would stall replication. *E. coli* and its relatives are unique in their acquisition of adenine methylation and SeqA function, perhaps allowing an extra level of control of replication and chromosome segregation, lacking in other bacteria.

### SeqA, Methylation, and Origin Dynamics

The origin region is particularly rich in GATC sites and SeqA binding to these sites is relatively long-lived, up to 1/3 of the cell cycle, when the sites remain hemimethylated [18],[23]. Using a mutant in many of the GATC sites near the origin, *oriC*m*3* [39], we addressed the role of the methylation pattern of *oriC* during the stringent response. Our data indicates that in this mutant stringent arrest is partially intact. Some arrest, as judged by flow cytometry for DNA content, was seen in this mutant upon serine hydroxamate treatment. During chronic exposure to ppGpp, this mutation relieved inhibition of colony formation only modestly, approximately 10-fold, relative to wild-type strains. This was in contrast to the Δ*seqA* strain, in which no evidence of replication arrest was apparent and which was 1,000-fold more efficient in colony formation during chronic exposure to ppGpp. It is possible that the *oriC*m*3* mutation only produces a partial loss of SeqA binding to the origin, although the first characterization of this mutant suggests that loss of sequestration at *oriC* is complete [39]. A recent study [31] shows that transient colocalization of sister origins in cells with overlapping replication cycles depends on SeqA in a manner that is not affected by *oriC*m3 but dependent on the property of SeqA for self-aggregation. It may be this mode of SeqA binding that is required for stringent arrest.

### Stringent Control of Chromosome Segregation

This study provides new evidence that late stages of chromosome replication or chromosome segregation can be regulated in response to environmental conditions. The nature of this arrest is intriguing and suggests possible mechanisms. We observed segregated *oriC* regions marked by GFP-ParB but only one half the predicted number of *ter* regions. In contrast, after cell cycle arrest by rifampicin and cephalexin, the number of observed *ter* foci is equivalent to those of *oriC*. Loss of *ter* cohesion may be a prerequisite for chromosome segregation and a point of regulation by the stringent response.

Interestingly, the nature of stringent arrest of chromosome segregation is very similar to the arrest seen in cells depleted of the GTPase ObgE, which is required for chromosome segregation and survival after treatment with replication inhibitors [49],[50]. Both the *Bacillus subtilis* Obg and *E. coli* ObgE bind ppGpp ([51]; Persky and Lovett, unpublished results); *E. coli* Obg (also known as CgtA) interacts with ppGpp synthetase/hydrolase SpoT [52] and CgtA may regulate SpoT hydrolase activity in *Vibrio cholerae* [53]. One possibility is that Obg controls ppGpp levels; alternatively, Obg may be required to license cell cycle progression in a manner that is inhibited by ppGpp binding.

Replication and chromosome segregation are concurrent processes in growing *E. coli* cells and loci segregate as they are duplicated. In some studies [54],[55], there appears to be a delay of segregation of *ter* relative to other regions of the chromosome, suggesting that there could be special factors that control its segregation. The 2∶1 ratio of *oriC* to *ter* foci may suggest a post-replication cohesion, either because regions near *ter* have not fully duplicated or because fully-replicated sister chromosomes are held together by DNA or protein linkages. Although the marker frequency analysis showing a 1∶1 *oriC/ter* ratio appears to support the latter explanation, we cannot rule out the possibility that short or heterogeneous unreplicated sequences in the *ter* region after ppGpp accumulation escape our detection. In any case, this “cohesion” may assist in the organization of chromosome segregation that will occur when cells are released from arrest with multiple chromosomes. We observed a rapid segregation of 4 nucleoids upon release, concomitant with nucleoid compaction, with segregation occurring first at midcell and later at the quarter position. Cohesion at the termini (Figure 5C) may restrain segregation of recent sister chromosomes until all others have segregated, a means of enforcing sequential patterns of segregation.

### Possible Mechanisms of Chromosome Segregation Control

In vitro, SeqA wraps DNA, producing positive supercoiling and can form cooperative self-aggregates [56]. This aggregative property, specifically altered in *seqA* N-terminal mutants, is implicated in the formation of visible foci colocalized to the replication fork and for promoting organization of the origin [31],[57]. The effects exerted by SeqA in origin-proximal cohesion could act in the same manner at the termini to promote their cohesion. We do note that, as *ter* is the last region to be replicated, its hemimethylated GATC sites should be bound by SeqA prior to segregation, giving the opportunity for SeqA to control separation and segregation of this region of the chromosome. Chromosome cohesion by prolonged binding during ppGpp accumulation could, in turn, signal a block to cell division.

Alternatively, apparent cohesion of sister chromosome could be mediated by DNA topological links. SeqA interacts with Topoisomerase IV, with the potential to modulate decatenation of the replicated chromosomes [58]. In vitro, at moderate levels of SeqA, decatenation and relaxation by Topo IV is stimulated by specific recruitment of the enzyme. At high levels, SeqA promotes catenane formation by Topo IV by promoting intermolecular aggregates. In either case, an increased probability of intertwined, catenated chromosomes upon induction of the stringent response could explain failure of the terminus regions of the chromosome to segregate after replication.

### Signal Transduction

Although our genetic analysis places SeqA and Dam in the cell cycle aspect of the stringent response, the direct connection between ppGpp, changes in the transcriptional program and modulation of SeqA or Dam methylase activity remains to be elucidated. Neither SeqA nor Dam bind guanine nucleotides; therefore some factor responsive to ppGpp is implicated in their control. An obvious candidate is RNA polymerase. Although transcription is required to initiate replication (and is therefore blocked by the RNA polymerase inhibitor, rifampicin), the mechanism of this control is still obscure, since promoter activity at *oriC* is not required for initiation under normal conditions [59]. We note that many of the strains defective in stringent control of replication, such as *seqA*, *dam*, *oriC*m*3* and *dam* overexpressors, may also exhibit rifampicin-resistant replication, as evident in run-out experiments, suggesting some connection between the two phenomena. An attractive model is that transcription sensitive to ppGpp may be required to reverse inhibitory effects of SeqA on replication initiation and chromosome segregation. This could be because SeqA binding blocks some DNA site required for cell cycle progression, with the act of transcription of this locus directly reversing this. Or alternatively, ppGpp-sensitive transcription of some unknown gene may be required to dissociate SeqA.

Previous reports have shown that DnaA expression decreases during the stringent response [35]. Although this may play some role in stringent control of replication initiation, this alone appears unlikely to mediate all stringent response effects on cell cycle. We were not able to suppress *seqA* defects in stringent arrest by *dnaA* mutants with reduced initiation efficiency. Moreover, DnaA is unlikely to directly influence chromosome segregation arrest during the stringent response. Nonetheless, it is possible that multiple mechanisms of cell cycle control, such as the DnaA levels and the regulatory inactivation of DnaA (RIDA), cooperate to control cell cycle in response to ppGpp.

## Materials and Methods

### Bacterial Strains and Growth Conditions

*Escherichia coli* K-12 strains (Table 2) were grown at 30°C, 34°C, 37°C as previously described on Luria-Bertani (LB) medium, consisting of 1% Bacto Tryptone, 0.5% yeast extract, 0.5% sodium chloride and, for plates, 1.5% agar or in M9 minimal medium (48 mM Na_2_HPO_4_-7H2O, 22 mM KH_2_PO_4_, 8.5 mM NaCl, 19 mM NH_4_Cl, 2 mM MgSO_4_ and 0.1 mM CalCl_2_) with 0.2% glucose, 0.4% glucose, or 0.4% arabinose, and 0.2% casamino acids with 1.5% agar for plates. For P1 transductions and phage lysates, cultures were grown in LCG, LB medium supplemented with 1% glucose with an additional 2 mM calcium chloride; for plates, 1% agar was added. Antibiotics were used in the following concentrations: ampicillin (Ap), 100 µg/ml; kanamycin (Km), 60 µg/ml; tetracycline (Tc) and chloramphenicol (Cm), 15 µg/ml. Isogenic strains in MG1655 were constructed by P1 vir*a* transduction. Cultures employed M9 minimal media described above with the addition of 1 mg/ml DL-Serine hydroxamate (Sigma) or, in the case of strains harboring pALS13 (pRelA′), 1 mM isopropyl β-D-1-thiogalactopyranoside IPTG for 1.5 hrs. Addition was made in early logarithmic growth of the culture, at OD_600_ of ∼0.2. Experiments examining chloramphenicol-induced translational inhibition were performed with the addition of 300 µg/ml chloramphenicol for 1.5 hrs. Release from DL-serine hydroxamate was accomplished by centrifugation of the treated culture and resuspension of the cell pellet in an equal volume of fresh medium without the drug. For experiments employing radioactive inorganic phosphate labeling, MOPS-buffered minimal medium was used (50 mM MOPS pH 7.2, 43 mM NaCl, 93 mM NH_4_Cl 1 mM MgSO_4_, 3.6 µM FeSO_4_-7H_2_O, 0.12 mM CaCl_2_ and either 2.2 mM or 0.4 mM KH_2_PO_4_).

**Table 2:** Strain and plasmid list.

A. Strain	Relevant Genotype	Source or Derivation
MG1655^a^	K-12 wild-type *F-rph-1*	[62]
STL 7210	*dam*::EZ-Tn5	[40]
STL 7222	*seqA*Δ::FRT *cat*	[40]
STL 7836	*seqA*Δ::FRT	
STL 8178	*relA*Δ::FRT *cat*	
STL 8525	*seqA*Δ::FRT *cat dnaA*(Sx)*721 zib*::Tn*10*	Cm^r^ transductant P1 STL7222×STL8404
STL 8783	*relA251*::*kan*	[63]
STL 8404	*dnaA*(Sx)*721 zib*::Tn*10*	[40]
STL 10173	*dam*::EZ-Tn5 [pALS13]	
STL 10175	*dam*::EZ-Tn5 [pALS14]	
STL 12758	*pstA*::P1*parS*	Km^r^ P1 transductant of CC4711×MG1655
STL 12760	*gadB*::P1*parS*	Km^r^ P1 transductant of CC4713×MG1655
STL 12787	*pstA*::P1*parS relA*Δ::FRT	Cm^r^ transductant P1 STL 8178×STL12758
STL 12858	*gadB*::P1*parS relA*Δ::FRT	Cm^r^ transductant P1 STL 8178×STL12760
STL 11505	*oriCm3*	[39]
STL 12701	*oriCm3 zid-501*::Tn*10*	Tc^r^ transductant P1 STL527×STL 11505
STL 8297	*dnaA46 tna*::Tn*10*	[49]
**B. Plasmid**		
pALS13	*relA*′	[38]
pALS14	*relA*Δ	[38]
pALA2705	GFP-Δ30::*parB*	[42]
pBAD18-*dam*	*bla araC dam^wt^*	This study^b^

a All strains are isogenic with MG1655 with the genotype F-*rph*-1.

b The *dam* gene was cloned using the Gateway Cloning Technology and methods provided by the manufacturer (Invitrogen). The *dam* gene was amplified by PCR with Triplemaster (Eppendorf), primers 5′-GGGGACAAGT TTGTACAAAA AAGCAGGCTT CACAGCCGGA GAAGGTGTAA TTAGTTAGTC AGCATGAAGA AAAA and 5′-GGGGACCACT TTGTACAAGA AAGCTGGGTT TATTTTTTCG CGGGTGAAAC GACT and the chromosomal template DNA derived from *E. coli* K-12 MG1655 (Masterpure DNA purification kit, Epicentre). After purification (Qiagen PCR purification kit), the *dam* gene was inserted into vector pDONR201 and subcloned from this plasmid into the destination vector pSTL360 [64], a derivative of pBAD18 [65] with an arabinose-regulated promoter. Plasmids were recovered by transformation into *E. coli* K-12 strain DH5α. The presence of intact *dam* in these plasmids was confirmed by DNA sequence analysis.

### Flow Cytometry

DNA content per cell was determined as described [49]. Briefly, 1 ml of culture was fixed in 9 mls of 70% ethanol and stored at 4°C until staining. For staining, fixed cultures were resuspended in 1 ml phosphate-buffered saline (PBS) pH 7.4. The samples were incubated with 100 µl PicoGreen dye (Invitrogen), diluted in 1∶100 in 25% DMSO for 3 hr at room temperature, and then further diluted with an additional 1 ml PBS containing PicoGreen (1∶1000). Cultures were analyzed by using a FACSCalibur flow cytometer and FloJo 6.4.1 software. As a control for chromosome number, a stationary phase wild type culture and an isogenic *dnaA46* strain, temperature sensitive for replication initiation, was analyzed similarly after 3 hr of growth at 42°C.

### Fluorescence Microscopy

Overnight cultures were inoculated into fresh medium at a dilution of 1∶100 and grown with aeration for 3 hours. Nucleoid staining was performed as previously described [28]. For DAPI staining alone, cultures were fixed in 3∶1 methanol acetic acid. 10–20 µl of cells was placed on poly-L-lysine hydrobromide (1 mg/ml)-coated slides and air-dried. Cells were washed 3 times with 1× Phosphate-Buffered-Saline (PBS [pH 7.4]) and allowed to air dry. Cells were then stained with 10 µl of 10 µg/ml DAPI (4′,6′ diamido-2-phenylindole) for 10 min and washed three times with PBS and mounted in 1 mg/ml p-phenylenediamine 90% glycerol in PBS, mounted with 5 µl VectaShield mounting medium and analyzed as described below. Living cells harboring pALA2705 (ParB-GFP) were grown at 34°C without supplementation of isopropyl β-D-1-thiogalactopyranoside (IPTG) to induce synthesis of the GFP fusions. A suspension of growing cells was added to a 2% agarose pad (MP Biomedicals, Inc) and covered with a cover slip over the residual media. Slides were analyzed with an Olympus BX51 microscope equipped with a RGB liquid crystal color filter. Images were acquired with a Qimaging Retiga Exi camera by using the manufacturer's software. Foci counts were obtained using Openlab Darkroom imaging software (Improvision, Coventry, United Kingdom) and edited with Openlab and Adobe Photoshop Elements 4.0.

### Marker Frequency Analysis Using Southern Hybridization

Cells were grown to exponential growth phase in M9 0.4% glucose CAA medium. Samples were taken and chromosomal DNA was extracted using the MasterPure DNA Purification Kit (Epicentre). Restriction digest and preparation of the probe were done as described previously [60]. Briefly, the chromosomal DNA was triple digested with *Eco*RI, *Hin*dIII, and *Eco*RV and the fragments were separated on a 1.0% agarose gel. The DNA was vacuum-transferred to a nylon membrane (Amersham). The membrane was prehybridized with BSA for more than 1 h at 65°C and hybridized overnight at 65°C with ^32^P a-dATP. The probe consisted of two DNA fragments that anneal to the chromosomal regions *gidA* (84.3 min), and *relB* (34.8 min). The DNA fragments were labeled using Random Primer Labeling (Molecular Cloning). After hybridization, the membrane was washed with 0.21 M Na_2_HPO_4_ (pH 7.3) / 6% SDS / 0.85 mM EDTA 3× for 10 minutes at 25°C followed by 2× for 5 minutes at 65°C. The membrane was exposed on a Phophoimaging screen (Molecular Dynamics) and scanned on a Bio–Rad Molecular Imager FX (Bio-Rad). Analysis of bands was carried out using Quantity One imaging software (Bio-Rad). Normalization of the bands was accomplished using genomic DNA from *dnaA46* that was grown at the non-permissive temperature for 2 h.

### Plating Efficiency of Cells Overexpressing RelA′

Overnight cultures grown in M9 CAA media were diluted 1∶100 in fresh media and grown to an OD_600_∼0.3. 10-fold serially diluted cultures were plated on M9 minimal media plates containing ampicillin and 100 µM IPTG. Total colony numbers were determined by plating on M9 ampicillin medium without IPTG. Colonies were counted after 48 hours of growth at 37°C. The number of independent cultures, as indicated in figure legends, was determined on at least three different days.

### Extraction and Pulse-Labeling of rRNA with [^3^H] Uridine

Overnight cultures were diluted 1/50 in M9 CAA media and grown to an O.D._600_∼0.2. Where indicated, cells were treated with serine hydroxamate. Pulse labeling was initiated by the addition of 20 µCi of [^3^H] uridine per 2 ml of culture. Uracil was added to 0.9 mg/ml after a ten minute pulse. Total RNA was extracted from 0.5 ml by the RNAeasy kit (Qiagen). Purified RNA was resuspended in RNase free water and stored at −20°C. Approximately 1 µg of RNA sample was analyzed by electrophoresis in a 1% non-denaturing agarose gel. After electrophoresis, the gel was photographed under UV light. Amount of rRNA was determined by band intensity of the 16S, 23S, and unprocessed rRNA bands in the image using Quantity Oneâ imaging software (Bio-Rad). To determine the amount of [^3^H] labeled rRNA, corresponding rRNA bands were cut out from the gel, dissolved, and analyzed in a scintillation counter. Normalization was achieved by dividing the obtained cpm by the volume of rRNA band intensity.

### Extraction and rRNA Labeling by ^32^P_i_ Incorporation

Overnight cultures grown in MOPS pH 7.2 medium containing 2 mM phosphate (KH_2_PO_4_) and 0.2% casamino acids were diluted 1∶100 into MOPS medium containing 0.4 mM phosphate and 0.2% casamino acids. Treated samples received 1 mg/ml SHX before the addition of [^32^P]H_3_PO_4_ to a final concentration of 200 µCi/ml when the culture OD_600_ reached ∼0.05. Total RNA was isolated after 15 minutes of incubation with [^32^P]H_3_PO_4_. RNA was isolated using the RiboPure Bacteria Kit from Ambion according to the manufacturer's instructions. Total RNA was electrophoresed on a native agarose gel and stained with ethidium bromide. rRNA bands were visualized and quantified by UV- light induced fluorescence using Quantity One software (Bio-Rad). RNA was then transferred to a nylon membrane (Amersham) using the Vacuum Blotter 785 (Bio-Rad). The amount of radiolabeled rRNA was determined by autoradiography and quantified using MolecularImager FX PhosphorImager and Quantity One software (Bio-Rad). Amount of radiolabeled rRNA was normalized to the amount of RNA observed by UV-light induced fluorescence.

### (p)ppGpp Measurements

(p)ppGpp measurements were performed as previously described [61]. Overnight cultures grown in MOPS minimal medium containing 0.4% Glucose 2 mM phosphate (KH_2_PO_4_) and 0.2% casamino acids were diluted 1∶100 into the same MOPS medium except containing 0.4 mM phosphate. [^32^P]H_3_PO_4_ was added to a final concentration of 100 µCi/ml when the OD_600_ reached ∼0.05 and cultures grew for an additional 3 hours before the first sample was taken. SHX was added at time 0, and samples were isolated every ten minutes by mixing 100 µl of culture with an equal volume of 13 M formic acid and chilling on dry ice. The samples were subjected to two rounds of freezing and thawing before microcentrifugation at 14, 000 rpm for 2 minutes to remove cellular debris. 6 µl of supernatant were spotted onto 20×20 cm polyethyleneimine cellulose on polyester TLC plates (Sigma) in 1.5 KH_2_PO_4_ (pH 3.4) for 2 h. After chromatography, nucleotides were visualized by autoradiography and quantified with a MolecularImager FX PhosphorImager and Quantity One software (Bio-Rad). Unlabeled GDP and GTP were spotted on the plates as markers and visualized after chromatography by UV light-induced fluorescence. The identities of the labeled (p)ppGpp were inferred from their positions in the chromatograph relative to the origin and GTP. (p)ppGpp levels are normalized to levels of GTP observed in the same sample.
